# Evaluation of the effect of surface moisture on dentinal tensile bond strength to dentine adhesive: An *in vitro* study

**DOI:** 10.4103/0972-0707.71640

**Published:** 2010

**Authors:** Thumu Jayaprakash, M R Srinivasan, R Indira

**Affiliations:** Department of Conservative Dentistry & Endodontics, Mamata Dental College, Khammam, Andhra Pradesh, India; 1 Department of Conservative Dentistry & Endodontics, Venkateswara Dental College, Chennai, India; 2 Department of Conservative Dentistry & Endodontics, Ragas Dental College, Chennai, India

**Keywords:** Adhesive, blot dry, dentine, surface moisture

## Abstract

**Aim:**

To evaluate the effect of surface moisture on dentinal tensile bond strength.

**Materials and Methods:**

Forty freshly extracted caries free, unrestored human mandibular molars were selected. The occlusal surfaces of each tooth were ground to prepare flat dentin surfaces at a depth of 1.5 mm. Following acid etching with 37% phosphoric acid for 15 sec, they were randomly grouped, with ten specimens in each: Group I – Over wet, Group II – Blot dry, Group III- One second dry, Group IV- Over dry. Each group was treated with a single bond adhesive system (3M ESPE) as per manufacturer’s instructions. Blocks or cylinders of composite resin were built up using Teflon mould and cured. Tensile bond strengths were tested using Instron universal testing machine. The results were statistically analyzed.

**Results:**

The mean tensile bond strength values of group II, Blot dry was highest and statistically significant (*P*<0.001).

**Conclusion:**

After acid etching and rinsing blot drying provided consistently better bond strength.

## INTRODUCTION

In 1955, Buonocore[1] proposed that acid etchants could be used to alter the enamel surface and make it more receptive to adhesion. On the other hand, dentinal adhesion has proved to be more difficult and less predictable. Much of the difficulty in bonding to dentin results from its structure, which is morphologically heterogeneous and physiologically dynamic. Dentinal adhesion is further complicated by the formation of the smear layer, which appears on the dentinal surface when the dentine is cut or ground.

Adhesive systems have undergone a great evolution with major improvements in dentinal bonding that occur when hydrophilic monomers and organic solvents were added to primers and adhesives, enabling their use on moist dentin substrate. When these adhesive systems are applied on a conditioned dentin, a hybrid layer composed of collagen fibrils and residual hydroxyapatite crystallites impregnated with the resin is formed. This hybrid layer provides much resistance and rigidity to the original mineralized dentin, and it is the main bonding mechanism of most current adhesive systems.[2]

The maintenance of a moist dentin surface after acid conditioning is of major importance for optimal development of a uniform hybrid layer. The organic solvents contained in the primers acts as water chasers, displacing water and carrying the resin monomers into the remineralized dentine favouring hybridization. On the other hand, if the etched dentine surface is desiccated the interaction of other elements doesn’t occur. The primer is deposited on the dentinal surface without diffusing into the underlying collagen network. However, the dentinal surface should not be over wet, as excess water can dilute the primer and render it less effective.[2]

Mohan B *et al*,[3] stated that dentin-bonding systems with water as a solvent are found to rehydrate the collapsed collagen. Acetone-based adhesives are found to compete with moisture, and the acetone carries the resin deep into the dentin.

The importance of the composition of adhesive systems and the drying method used in the bonding procedures are evident, as they favor the formation of a homogeneous and uniform hybrid layer, enabling an effective bond between dentin and restorative materials. The aim of this study was to evaluate the effect of surface moisture on dentinal tensile bond strength.

## MATERIALS AND METHODS

Forty freshly extracted caries free, unrestored human mandibular molars were selected. The occlusal surface was ground until all occlusal enamel was removed. This resulted in exposure of flat dentine surface with enamel at periphery. The teeth were embedded into the self cure acrylic resin coated with nail polish of different colors for each group leaving the dentin surface exposed. The specimens were randomly divided into four groups of 10 teeth each. The occlusal surface of specimens was treated with 37% orthophosphoric acid for 15 sec and rinsed with water for 10 sec.

Group I - Over Wet: The excess water was not removed and it remained on the demineralized dentin surface.

Group II - Blot Dry: The excess water was blotted with tissue paper maintaining a moist dentine surface.

Group III - One Second Dry: The etched dentine surface was air dried for 1 secusing an oil free air source (Hair drier) from a distance of 10 cm.

Group IV - Over Dry: The etched dentin surface was air dried for 30 sec using compressed oil free air source (Hair drier) from a distance of 10 cm.

Single bond adhesive (3M) was applied to the demineralized dentin using a fully saturated brush tip of adhesive for each coat, two consecutive coats were applied and then gently air dried for 2-5 sec and light cured for 10 sec.

The composite resin was condensed to 2 mm thickness in a hollow poly vinyl cylinder and light cured for 40 sec. Another composite resin of 2 mm thickness was placed over the first placed composite increment. A 26 gauge ligature wire was twisted at one end and a loop was formed at another end. Twisted end was placed inside the 2 mm of uncured composite resin. The composite resin was then light cured for 40 sec. Following complete curing poly vinyl cylinder moulds were cut and removed, leaving the 4 mm of resin with ligature wire bonded to dentin. All the specimens were immersed in water for 24 h. Tensile bond strength was then measured using an Instron universal testing machine with a cross head speed of 0.5 mm per min.

## RESULTS

Results were subjected to statistical analysis (Kruskal Wallis one way ANOVA) and comparison between the groups was done by post-hoc. The mean tensile bond strength values of group II was the highest and was statistically significant, with group I, III and IV. Group II showed slightly higher values than group III. Group I showed the least value among the groups tested. The mean tensile bond strength and standard deviation values are presented in Table 1.

**Table 1:** The mean tensile bond strength and standard deviation values of different groups

One way anova				
	N	Mean	Std. Deviation	Std. Error
Group I	10	8.2470	8.526E-02	696E-02
Group II	10	21.778	3.205E-02	014E-02
Group III	10	20.464	5.773E-02	826E-02
Group IV	10	9.602	6.378E-02	017E-02
Total	40	15.023	6.2133	.9824

N - number of samples

Table 2 shows the statistical analysis of difference in tensile bond strength in between groups.

**Table 2:** Comparison between groups

Anova					
	Sum of Squares	Df	Mean Square	F	Sig
Between groups	1505.459	3	501.820	127883.0	.000
Within groups	.141	36	3.924E-03		
Total	1505.601	39			

Df - mean difference

## DISCUSSION

Adhesion of restorative material to tooth structure becomes a routine and reliable aspect of modern dentistry. Strong durable bonds between dental biomaterials and tooth structures are essential not only from a mechanical point of view, but also from biologic and esthetic consideration. In 1955, Buonocore postulated that acid could be used as a surface treatment before application of resins. In the late 1960’s, Buonocore suggested that it was the formation of resin tags that caused the principle adhesion of the resin to acid etched enamel. Fusayama in 1979 introduced the total etch concept which involves both enamel and dentin.

Kanca[4–7] and Gwinnet[8–11] recommended that etched dentin need not be dried before application of the bonding primers. They reported good success of ’moist bonding’ with acetone-based systems containing bisphenyl dimethacrylate (BPDM). Kanca[7] found a statistically significant difference in bond strength between dentin dried for 3 sec, 10 sec and blotted with a damp tissue prior to placement of the dentin primer. Since the advent of the ‘wet bonding’ protocol, numerous studies have been reported on improvements in bond strength with the use of wet compared to dry bonding methods, particularly with acetone and/ or ethanol containing adhesives.

Kimochi *et al*,[12] in 1999 suggested that 37% phosphoric acid to be used in order to attain high tensile bond strength. Thomas Pioch *et al*,[13] in 1998 had observed that the highest tensile bond strengths were achieved after 15’s of etching compared to 30’s and 60’s of etching. John Kanca[7] in 1996 suggested that air syringe-to-tooth distance of 10 cms gives the high tensile bond strengths. Eick and Walch[14] in 1986 suggested that applying the composite resin in increments of 2 mm could reduce polymerization shrinkage stresses.

Results of the study showed that Group II, Blot dry produced highest tensile bond strength compared to other groups. Values obtained were statistically significant between all groups. In the case of blot drying, after etching and blotting most of the water in the interfibrillar spaces remained to support the collagen network. During application of the adhesive, the ethanol solvent could drive water away sufficiently to allow penetration of the monomers. Thus, the monomers infiltrated into the demineralized dentin, polymerize and produce a hybrid layer without collapse of the collagen network or separation of the resin from the dentin.[15]

Kanca[6] and Gwinnett[8] suggested that resin primer solvents such as ethanol or acetone aided in the displacement of surface moisture, thus carrying the dissolved resin into the patent dentinal tubules and the demineralized intertubular dentin following acid conditioning. The rationale of wet or moist bonding is to prevent the collagen network exposed by acid etching from collapsing. Collapse of collagen fibrils occurs when the conditioned dentin surface is dried, thereby inhibiting infiltration of most current monomers into dentine.[10]

The wet bonding technique can promote efficient resin monomer diffusion into the demineralized matrix only if excess water on the dentin surface is eliminated with the dry cotton after etching, water rinsing, and then replaced by monomers during the subsequent step. In the currently available adhesive systems, hydrophilic primer monomers are dissolved in volatile solvents, such as acetone and ethanol, that aid in displacement of the remaining water and also carrying the polymerizable hydrophilic monomers into the opened dentinal tubules and through the nano-spaces of the collagen web[16].

According to Tay *et al*,[17] the presence of excess water dilutes the primer and its components into different phases. This leads to the formation of water blisters and micelles. Intratubular globules located below resin plugs could be formed when the dentin tubules were partially filled with water, at a site within the tubules where the ethanol could not displace the water any further.

Air drying for 1 sec resulted in partial collapse of the demineralized collagen matrices. However, since an ethanol- based adhesive invariably contains a small amount of water, this extrinsic water together with the increase in intrinsic moisture caused by removal of the smear layer could have resulted in rehydration of the partially collapsed collagen matrix during the 10 sec of adhesive application.[18]

When the dentine surface is dried, there is no water to chase and the wetting of the surface is diminished. The addition of alcohol to water decreases the surface tension of water, yet the bond strength generated was not as high as with the acetone solvent. This is evidently a consequence of alcohol having greater hydrogen bonding than acetone and thus it does not chase water as effectively. Again when the surface is dry, there is no interaction of the solvent with water and adhesion is diminished.[19]

## CONCLUSION

The results of this study have shown that blot drying with a tissue paper provided consistently better bond strength as compared to over wet, over dry and one second dry groups.

Future research concerning the effect of dentine moisture on the performance of current generation dentine bonding agents should focus on two areas. First attempts should be made to standardize the amount of moisture present on dentine immediately prior to bonding. Being able to quantify and standardize the amount of water present on the dentine surface studies by future researchers may lead to a better understanding of the effects of varying degrees of moisture on the performance of bonding agents. Future research should evaluate the effects of dentine moisture on 1-year and 3-year bond strength.
